# RACIAL RESIDENTIAL SEGREGATION AND COGNITIVE DECLINE AMONG OLDER ADULTS

**DOI:** 10.1093/geroni/igz038.101

**Published:** 2019-11-08

**Authors:** Joy Bohyun Jang, Margaret T Hicken, Philippa J Clarke, Ketlyne Sol, Robert Melendez, Min Hee Kim, Megan Mullins, Suzanne Judd

**Affiliations:** 1 University of Michigan, Ann Arbor, Michigan, United States; 2 University of Alabama at Birmingham, Birmingham, Alabama, United States

## Abstract

Racial residential segregation may be a fundamental cause of health disparities in the U.S., and few studies employ objective measures of segregation to estimate its impacts on cognitive decline. Using data from 21,446 REGARDS participants in urban areas, we employed race-stratified growth curve models to examine how city racial segregation was associated with trajectories of cognitive decline over time. Controlling for demographics and health conditions/behaviors, higher segregation for blacks was marginally associated with lower cognitive function at baseline (b=-0.159, p<.10) while higher segregation for whites was associated with better cognitive function (b=0.158, p<.01). For both blacks and whites, there were no significant associations between segregation and rate of cognitive decline but neighborhood poverty was adversely related to cognitive function (b=-0.171, p<.01 for blacks, b=-0.289, p<.001 for whites). Further research into mechanisms that contributes to heterogeneity in associations between racial segregation and cognitive function is needed to develop effective prevention interventions.

